# Subinhibitory Concentrations of Perilla Oil Affect the Expression of Secreted Virulence Factor Genes in *Staphylococcus aureus*

**DOI:** 10.1371/journal.pone.0016160

**Published:** 2011-01-19

**Authors:** Jiazhang Qiu, Xiaoran Zhang, Mingjing Luo, Hongen Li, Jing Dong, Jianfeng Wang, Bingfeng Leng, Xiaoliang Wang, Haihua Feng, Wenzhi Ren, Xuming Deng

**Affiliations:** 1 Key Laboratory of Zoonosis, Ministry of Education, Institute of Zoonosis, College of Animal Science and Veterinary Medicine, Jilin University, Changchun, People's Republic of China; 2 College of Chemistry, Jilin University, Changchun, People's Republic of China; Cairo University, Egypt

## Abstract

**Background:**

The pathogenicity of *staphylococcus aureus* is dependent largely upon its ability to secrete a number of virulence factors, therefore, anti-virulence strategy to combat *S. aureus*-mediated infections is now gaining great interest. It is widely recognized that some plant essential oils could affect the production of staphylococcal exotoxins when used at subinhibitory concentrations. Perilla [*Perilla frutescens* (L.) Britton], a natural medicine found in eastern Asia, is primarily used as both a medicinal and culinary herb. Its essential oil (perilla oil) has been previously demonstrated to be active against *S. aureus*. However, there are no data on the influence of perilla oil on the production of *S. aureus* exotoxins.

**Methodology/Principal Findings:**

A broth microdilution method was used to determine the minimum inhibitory concentrations (MICs) of perilla oil against *S. aureus* strains. Hemolysis, tumour necrosis factor (TNF) release, Western blot, and real-time RT-PCR assays were performed to evaluate the effects of subinhibitory concentrations of perilla oil on exotoxins production in *S. aureus*. The data presented here show that perilla oil dose-dependently decreased the production of α-toxin, enterotoxins A and B (the major staphylococcal enterotoxins), and toxic shock syndrome toxin 1 (TSST-1) in both methicillin-sensitive *S. aureus* (MSSA) and methicillin-resistant *S. aureus* (MRSA).

**Conclusions/Significance:**

The production of α-toxin, SEA, SEB, and TSST-1 in *S. aureus* was decreased by perilla oil. These data suggest that perilla oil may be useful for the treatment of *S. aureus* infections when used in combination with β-lactam antibiotics, which can increase exotoxins production by *S. aureus* at subinhibitory concentrations. Furthermore, perilla oil could be rationally applied in food systems as a novel food preservative both to inhibit the growth of *S. aureus* and to repress the production of exotoxins, particularly staphylococcal enterotoxins.

## Introduction

*Staphylococcus aureus* is a gram-positive pathogen that causes an array of diseases, ranging from minor localized skin lesions to life-threatening deep tissue damage and systemic infections such as pneumonia, endocarditis, and exotoxin syndromes [1]. Additionally, *S. aureus* is an important food-borne pathogen that causes staphylococcal gastroenteritis and food poisoning in humans [2]. *S. aureus* has the potential to produce a large number of secreted and cell wall-related virulence factors that contribute to the diversity of *S. aureus*-associated illnesses [3].

α-toxin, enterotoxins, and toxic shock syndrome toxin 1 (TSST-1) are among the major exotoxins secreted by *S. aureus*. α-toxin is a 33 kDa pore-forming extracellular protein that is secreted by most *S. aureus* strains and has hemolytic, cytotoxic, dermonecrotic and toxic properties. Penetration of host cell membranes by α-toxin leads to the formation of a hexameric transmembrane pore that causes cell lysis [4]. Many human cells, including erythrocytes, monocytes, lymphocytes, macrophages and epithelial cells, are affected by α-toxin. Staphylococcal enterotoxins (SEs) and TSST-1 are intermediate molecular weight proteins (20–30 kDa) that cause food poisoning and toxic syndrome, respectively, in humans and other species [5]. Moreover, both SEs and TSST-1 can act as bacterial superantigens that bind to MHC class II molecules on antigen presenting cells and selectively stimulate T cells expressing appropriate Vβ gene segments on their T cell receptors (TCRs) [6]. The secretion of staphylococcal exotoxins is regulated in a growth-phase-dependent manner, predominantly occurring during the post-exponential phase [7]. The accessory gene regulator (*agr*) locus, a well-characterized two-component regulatory system, plays a critical role in the regulation of exotoxin production by *S. aureus* [3].

The rapid development of antibiotic resistant strains has made it difficult to treat *S. aureus* infections. Therefore, both in the food and pharmaceutical industries, there is a continuous need to develop new therapeutics to aid in the prevention and treatment of lethal infections caused by these strains. Recently, much research has focused on plant extracts, in particular essential oils, due to their potent antimicrobial properties against a broad spectrum of microorganisms [4].

Perilla [*Perilla frutescens* (L.) Britton], a natural medicine found in eastern Asia, is primarily used as both a medicinal and culinary herb [8]. Its essential oil (perilla oil), which contains a high amount of α-linolenic acid (ALA), has been shown to possess a variety of medicinal properties, including antitumor, antioxidative, antimicrobial, anti-inflammatory, and anti-anaphylactic shock activities [9]–[13]. Furthermore, nutritional studies have shown that perilla oil is beneficial for healthy aging and learning performance [14], [15]. The objective of this study was to assess the anti-*S. aureus* activity of perilla oil and to further investigate the influence of subinhibitory concentrations of perilla oil on the production of α-toxin, the two major enterotoxins (SEA and SEB), and TSST-1 in both methicillin-sensitive *S. aureus* (MSSA) and methicillin-resistant *S. aureus* (MRSA).

## Results

### Impact of perilla oil on *S. aureus* growth

In this study, perilla oil exhibited a significant antibacterial activity against the test strains, with MIC values of 0.2–0.8 µl/ml. The MIC values of perilla oil against *S. aureus* strains ATCC 29213, MRSA 2985 and MRSA 3701, which were selected for further experiments, were all 0.4 µl/ml.

**Figure 1:**
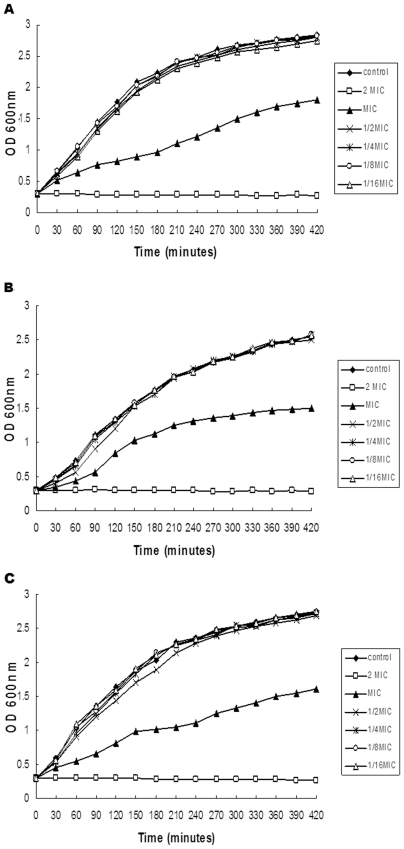
Growth curves of *S. aureus* after exposure to various concentrations of perilla oil. *S. aureus* strains ATCC 29213 (A), MRSA 2985 (B), and MRSA 3701 (C) were grown in the presence of increasing concentrations of perilla oil at 37°C with agitation at 200 rpm under aerobic conditions, and cell growth was monitored by measuring the OD values at 600 nm at the indicated time points. These curves represent one of three reproducible experiments.

Prior to evaluating the influence of subinhibitory concentrations of perilla oil on the production of bacterial virulence factors, we first investigated the effect of increasing concentrations of perilla oil on the growth of *S. aureus*. As shown in Fig. 1A, perilla oil, at concentrations ranging from 1/16 to 1/2× MIC, had no significant influence on the growth of *S. aureus* strain ATCC 29213. However, treatment with MIC of perilla oil could inhibit the growth rate; after 60, 210 and 420 min of perilla oil treatment, the OD_600 nm_ values were 62.1%, 45.6% and 63.6% of the perilla oil-free culture, respectively. Notably, when cultured with 2× MIC of perilla oil, the growth of *S. aureus* was completely inhibited. Although the growth kinetics can vary greatly between strains, the growth of MRSA 2985 and MRSA 3701 were affected in a similar manner by these concentrations of perilla oil. In other words, supplementation with 1/16, 1/8, 1/4, and 1/2× MIC of perilla oil had no significant effects on MRSA 2985 and MRSA 3701 growth (Fig. 1B & C.).

Moreover, the relationship between the OD values and colony-forming units (CFUs) was determined by viable counts. The data indicated that the levels of bacteria in the control culture and the perilla oil-treated (1/16, 1/8, 1/4, and 1/2× MIC) cultures were approximately 1.0×10^9^ CFU/ml when grown to an OD_600 nm_ value of 2.5. Furthermore, no morphological differences were observed when *S. aureus* ATCC 29213 cultured with graded subinhibitory concentrations of perilla oil to the post-exponential phase (Fig. S1).

### Perilla oil decreases hemolytic and T-cell stimulating activities of *S. aureus* culture supernatants

Hemolysins secreted by *S. aureus* can promote hemolysis of rabbit erythrocytes, while other proteins secreted by the bacteria can act as superantigens which stimulate T-cell proliferation. Therefore, a hemolysis assay and a murine T-cell proliferation assay were used to investigate the effect of perilla oil treatment on the hemolytic and T-cell stimulating activities of *S. aureus* culture supernatants.

As shown in Table 1, when supplemented with 1/16× MIC of perilla oil, the hemolytic activities of the culture supernatants of *S. aureus* strains ATCC 29213, MRSA 2985 and MRSA 3701 were 82.1%, 48.3% and 77.8% of their drug-free culture, respectively. Remarkably, no hemolytic activity was observed when cultures were grown in the presence of 1/2 or 1/4× MIC of perilla oil. This dose-dependent inhibition of hemolysis was observed in all of the investigated strains. Additionally, perilla oil itself did not induce or inhibit hemolysis of rabbit erythrocytes at 1× MIC, and there was little influence on the hemolytic activity of culture supernatants when pre-incubated with 1× MIC of perilla oil (data not shown). Cells of *S. aureus* cultures in the presence of 1/2× MIC of perilla oil were collected and re-grown in fresh MHB to the post-exponential phase. The hemolytic activity of the culture supernatants was evaluated. The results indicated that the inhibited hemolytic activity was reversible. Therefore, the reduction is not likely to be due to the selection of mutants.

**Table 1:** Hemolytic activities of *S. aureus* culture supernatants treated with graded subinhibitory concentrations of perilla oil.

Strains	Hemolysis (%) of rabbit erythrocytes by culture supernatant^a^					
	0	1/16× MIC	1/8× MIC	1/4× MIC	1/2× MIC	Re-grown cultures^c^
ATCC 29213	100%	82.1%±7.9	42.5%±6.2*	NO^b^	NO.	94.3%±6.6
MRSA 2985	100%	48.3%±3.5*	12.5%±4.1**	NO.	NO.	98.2%±4.9
MRSA 3701	100%	77.8%±6.8	54.1%±5.2	29.1%±3.9**	NO.	90.6%±7.8

a The drug-free culture supernatants served as the 100% hemolysis control.

b NO. signifies that there was no observed hemolytic activity.

c Cells of *S. aureus* in the presence of 1/2× MIC of perilla oil were harvested and re-grown in fresh MHB.

Values represent the mean and standard deviation of three independent experiments. * indicates *p* <0.05 and ** indicates *p*<0.01, when compared to the corresponding control.

**Figure 2:**
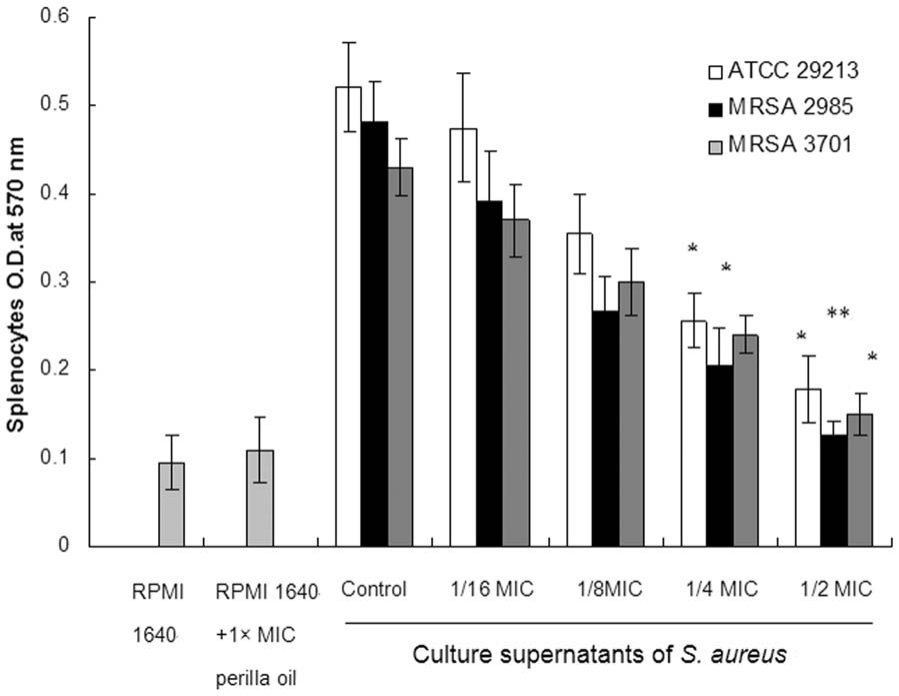
Induction of murine splenocyte proliferation by *S. aureus* supernatants. *S. aureus* strains were cultured with graded subinhibitory concentrations of perilla oil in RPMI 1640. Values represent the mean ± SD for three independent experiments. * represents *p*<0.05 and ** represents *p*<0.01.

As shown in Fig. 2, the culture supernatants of *S. aureus* grown in the presence of increasing concentrations of perilla oil showed a significantly reduced stimulatory effect on T-cell proliferation. Additionally, perilla oil itself did not stimulate or inhibit T-cell activation at 1× MIC. Perilla oil appeared to attenuate the T-cell-activating activity of culture supernatants in a dose-dependent fashion.

### Perilla oil decreases α-toxin, SEA, SEB, and TSST-1 levels in *S. aureus* culture supernatants

The addition of 1/16, 1/8, and 1/4× MIC of perilla oil has no significant influence on *S. aureus* extracellular protein concentration. However, a slight decrease of protein secretion was observed when cultured with 1/2× MIC of perilla oil (Table 2).

**Table 2:** Protein contents in supernatants of *S. aureus*.

Strains	Extracellular protein concentration (mg/ml) of *S. aureus* after treatment with perilla oil a				
	0	1/16× MIC	1/8× MIC	1/4× MIC	1/2× MIC
ATCC 29213	7.521±0.52	7.562±0.46	7.389±0.23	7.435±0.44	6.009±0.36
MRSA 2985	6.453±0.38	6.278±0.22	6.477±0.35	6.199±0.34	5.554±0.28
MRSA 3701	5.794±0.66	5.651±0.41	5.468±0.55	5.712±0.37	4.812±0.49

a Mean values ±SD from three independent experiments.

**Figure 3:**
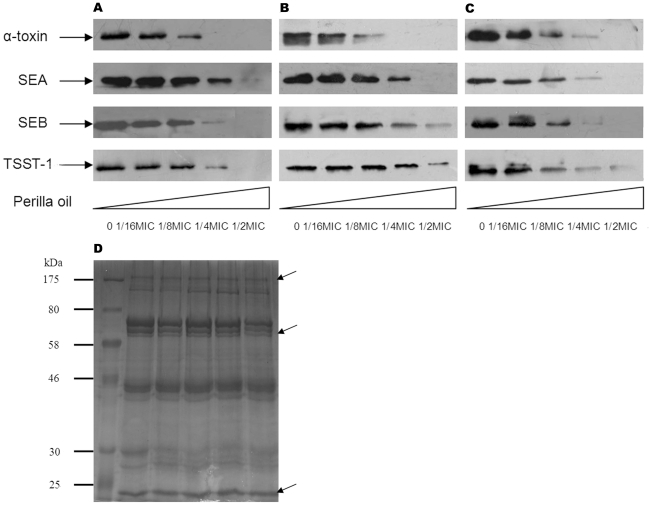
Western blot analysis of α-toxin, SEA, SEB, and TSST-1 production. *S. aureus* strains ATCC 29213 (A), MRSA 2985 (B) and MRSA 3701 (C) were grown in the presence or absence of increasing concentrations of perilla oil in MHB until the post-exponential growth phase. Equal amount of extracellular proteins were subjected to western blot analysis. (D) Coomassie-stained SDS-PAGE of secreted proteins by *S. aureus* strain ATCC 29213, arrows indicate the proteins that are not influenced by perilla oil.

Among the secreted proteins of *S. aureus*, α-toxin is the exotoxin primarily responsible for the hemolytic activity of *S. aureus* culture fluid, while SEs and TSST-1 are the most critical exotoxins that can act as superantigens, stimulating T-cell-activation. Western blot analysis was performed to determine whether the reduced hemolytic and T-cell stimulating activities of *S. aureus* culture supernatants cultured in the presence of increasing concentrations of perilla oil was due to the diminished production of α-toxin, SEA and SEB (the major SEs), and TSST-1. As shown in Fig. 3, treatment with graded subinhibitory concentrations of perilla oil resulted in a dose-dependent decrease in the production of α-toxin, SEA, SEB, and TSST-1. Growth with 1/16× MIC of perilla oil resulted in a recognizable reduction in the secretion of these toxins; while at 1/2 or 1/4× MIC, little to none of these immunoreactive proteins could be detected in the culture fluids of any of the strains tested.

**Figure 4:**
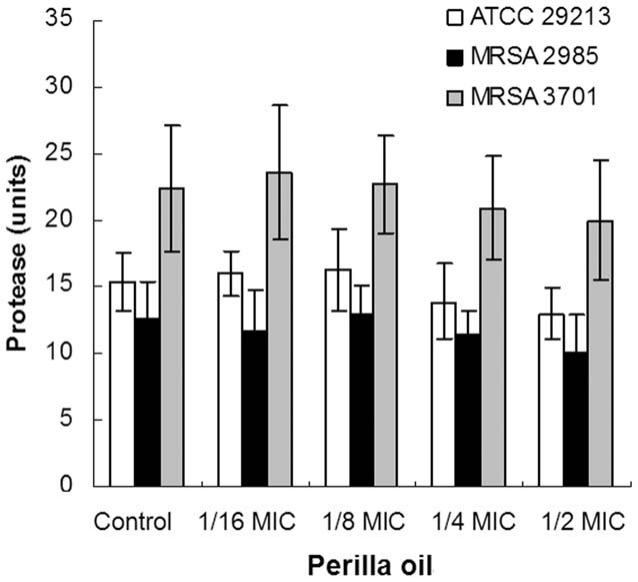
Protease units of *S. aureus* culture supernatants. Bacteria were cultured with increasing concentrations of perilla oil to reach an OD_600nm_ of 2.5. The culture supernatants were collected by centrifugation, and used for proteolytic activity assay. Values represent the mean ± SD for three independent experiments.

The proteolytic activity of the culture supernantants was determined to confirm whether the reduction of toxins production in *S. aureus* was due to the increase in protease secretion induced by perilla oil. There was no significant influence on protease secretion by ATCC 29213, MRSA 2985 or MRSA 3701 cultured with subinhibitory concentrations of perilla oil (Fig. 4).

### Perilla oil inhibits the transcription of *hla, sea, seb, tst,* and *agrA* in *S. aureus*

Based on the finding that perilla oil significantly represses the production of α-toxin, SEA and SEB, and TSST-1 in *S. aureus*, we further used a real-time RT-PCR assay to investigate the relative expression levels of these toxin-encoding genes (*hla*, *sea*, *seb*, and *tst*, respectively) after treatment with perilla oil. Additionally, because the expression of these genes is positively regulated by the *agr* locus [16], the transcription level of *agrA* was also detected. As expected, the expression levels of these genes in *S. aureus* strain ATCC 29213 were markedly repressed upon treatment with perilla oil (Table 3). When cultured with 1/2× MIC of perilla oil, the expression levels of *hla*, *sea*, *seb*, *tst* and *agrA* were decreased by 10.7-, 6.1-, 5.5-, 9.5- and 6.4-fold, respectively.

**Table 3:** Relative gene expression levels of *hla*, *sea*, *seb*, *tst* and *agrA* in *S. aureus* ATCC 29213 after treatment with 0.2 µL mL^−1^ of perilla oil.

Gene	Product	Fold changes ± SD^a^
*hla*	α-toxin	−10.7±2.7
*sea*	Staphylococcal enterotoxin A	−6.1±1.8
*seb*	Staphylococcal enterotoxin B	−5.5±1.5
*tst*	Toxic shock syndrome toxin 1	−9.5±2.4
*agrA*	Accessory gene regulator A	−6.4±2.0

a The real-time RT-PCR results represent the mean of three biological replicates with three technical replicates for each gene. SD indicates standard deviation.

## Discussion

The emergence of multidrug-resistant strains of *S. aureus* presents a threat to the worldwide population; therefore, there is a continuing quest to discover new antimicrobial compounds from unexplored sources [17]. Recently, plant extracts, and in particular their essential oils, have garnered great interest for their potent antimicrobial properties against a broad spectrum of microorganisms [18]. In the last few years, numerous studies have been conducted in different countries to demonstrate their usefulness in therapeutic treatments [19], [20]. Perilla [*Perilla frutescens* (L.) Britton] is a medicinal and edible plant of the family Labiatae. In indigenous medicine, perilla has long been used to treat colds, headaches, and coughs. Its essential oil (perilla oil) has been shown to possess a number of pharmacological activities [9]–[13]. In this report, we confirmed that perilla oil was active against both MSSA and MRSA with MICs ranging from 0.2–0.8 µl/ml. Therefore, perilla oil may be useful as a potential antibacterial agent to combat the pathogen, and it may deserve further investigation for its potential therapeutic efficacy in *S. aureus* infections.

The pathogenicity of *S. aureus* is dependent, to a great extent, upon the secretion of a variety of extracellular virulence factors [3]. Therefore, the clinical performance of antimicrobial agents used for the treatment of *S. aureus* infections not only depends on the respective bacteriostatic or bactericidal effects, but also on the ability to prevent the release of virulence factors by dying or stressed bacteria [7]. Additionally, an alternative therapeutic strategy for *S. aureus* infections that is now gaining great interest is the targeting of bacterial virulence, which provides promising opportunities to abate pathogenicity and its consequences without exerting immediate life-or-death pressure on the target pathogen [21].

It has long been known that many antibiotics that exhibit little or no influence on overall growth can affect the expression of *S. aureus* virulence factors when used at sub-lethal concentrations. The antibiotic-induced regulation of virulence factors may result in either aggravation or attenuation of the infection. Therefore, the potential of antibiotics to affect these properties may be an important consideration when selecting an antibiotic for therapy. For instance, protein-synthesis-inhibiting antibiotics such as clindamycin, linezolid, and quinupristin/dalfopristin are recommended for the treatment of *S. aureus*-induced toxic syndromes because concentrations below the MIC have been shown to decrease the production of exotoxins in *S. aureus* (including α-toxin, SEA, SEB, TSST-1, and protein A) [7], [22], [23]. In contrast, β-lactam antibiotics proved unfavorable for the management of toxin-related *S. aureus* infections, as even subinhibitory concentrations (for example of methicillin) promote an increase in α-toxin and TSST-1 production via a stimulatory effect on exoprotein synthesis [24]. In this report, through the use of transcriptional, expressional and phenotypic analyses, we have shown that subinhibitory concentrations of perilla oil dose-dependently suppress α-toxin, SEA and SEB, and TSST-1 expression in both MSSA and MRSA. These data suggest that perilla oil may be useful for the treatment of *S. aureus* infections when used in combination with β-lactam antibiotics, which can increase exotoxin production by *S. aureus* at subinhibitory concentrations.

In food industry a negative consumer reaction to chemical preservatives has prompted an increased interest in natural alternatives [25]. For these reasons, plant essential oils, because of their established antimicrobial activities as well as their relatively lower toxicity and reduced number of side effects, may be potentially useful replacements for chemical preservatives [18]. Because plant essential oils have a multi-component nature, it is more difficult for bacteria to develop resistance than many common used antibiotics, which have a single target site [26]. Additionally, staphylococcal food poisoning does not result from the ingestion of *S. aureus* itself, but rather from ingestion of one or more preformed staphylococcal enterotoxins in food that has been contaminated with the bacteria [27]. Therefore, considering its anti-*S. aureus* activity, as well as the findings that it significantly decreases the production of SEA and SEB by *S. aureus*, perilla oil shows promise as a novel food preservative both to inhibit the growth of *S. aureus* and to suppress the expression of SEs.

In *S. aureus*, the production of virulence factors during the growth cycle is controlled by global regulators such as *agr* and SarA [16]. Previous studies have shown that subinhibitory concentrations of antibiotics can affect the translation of certain regulatory gene products in *S. aureus,* which, in turn, alter the transcription of toxin-encoding genes. For instance, subinhibitory concentrations of thymol decreased exotoxin production in *S. aureus,* possibly in part due to inhibition of the *agr* locus [28]. Similarly, subinhibitory concentrations of clindamycin differentially reduced the transcription of exoprotein genes in *S. aureus*, in part through modulation of the *sar* locus [22]. To address this, a real-time RT-PCR assay was used to assess the impact of perilla oil on the expression level of the *agr* locus in *S. aureus*, which is thought to positively regulate α-toxin, SEA and SEB, and TSST-1 production [16]. The data indicated that the expression level of *agrA* was significantly reduced when *S. aureus* strain ATCC 29213 was cultured with 1/2× MIC of perilla oil. Nevertheless, with the current data, it is unknown whether *agr* or other regulators are involved. Therefore, we presume that the reduced production of these toxins may, in part, depend on inhibition of the *agr* locus induced by perilla oil.

## Materials and Methods

### Ethics Statement

All animal studies were conducted according to the experimental practices and standards approved by the Animal Welfare and Research Ethics Committee at Jilin University (Approval ID: 20100305-2). The study was also approved by the Institutional Review Board of the First Hospital of Jilin University, and all patients provided written informed consent for the collection of samples and subsequent analysis [29].

### Bacterial strains and reagents

The methicillin-sensitive *S. aureus* strain ATCC 29213 was obtained from American Type Culture Collection (ATCC). Twenty-six *S. aureus* isolates (7 MSSA and 19 MRSA) were acquired from clinical samples at the First Hospital of Jilin University [29]; the clinical MRSA strains 2985 and 3701, which have the potency to secrete α-toxin, SEA, SEB, and TSST-1, were used for further experiments. Bacteria were stored as 30% glycerol stocks at −80°C and subcultured on blood agar plates at 37°C before testing. Perilla oil (pure essential oil) was extracted by water distillation and supplied by the National Institute for the Control of Pharmaceutical and Biological Products (Beijing, China).

### MIC determination

The minimum inhibitory concentrations (MICs) of perilla oil against *S. aureus* strains were determined using a broth microdilution method as described by Carson et al. [30] with minor modifications. Stock solutions of perilla oil at various concentrations were prepared in dimethyl sulfoxide (DMSO) (Sigma-Aldrich, St. Louis, MO, USA). All tests were performed in Mueller–Hinton broth (MHB) (BD Biosciences, Inc., MD, USA) supplemented with Tween-80 (Sigma-Aldrich) at a final concentration of 0.5%. Serial doubling dilutions of perilla oil were prepared in a 96-well plate over the concentration range of 0.05–3.2 µl/ml. Following inoculation of 5×10^5^ cfu/ml of overnight broth cultures in each well, the plates were incubated aerobically at 37°C for 24 h. The MIC was defined as the lowest concentration of perilla oil at which the microorganism did not demonstrate visible growth.

### Growth curves and viable counts

Bacteria were grown in the presence of increasing concentrations of perilla oil at 37°C with agitation at 200 rpm under aerobic conditions, and cell growth was monitored by reading the OD values at 600 nm at the indicated time points.

When the OD_600 nm_ values were reached 2.5, serial 10-fold dilutions of the samples were spread onto drug-free Mueller-Hinton agar plates. The number of colonies was determined after an incubation period of 24 h at 37°C.

### Hemolysis assay

Total hemolysis was determined as described previously [28] with rabbit erythrocytes, which are 1000 times more sensitive to *S. aureus* α-toxin than human erythrocytes. In brief, bacteria were grown in MHB supplemented with 0.5% Tween-80 at 37°C with graded subinhibitory concentrations of perilla oil until reaching the post-exponential growth phase (OD_600 nm_ of 2.5). Culture supernatants were harvested and filter sterilized with a 0.22 µm pore-size acetate syringe filter. A 0.1 ml volume of culture supernatant was combined with 2.5% defibrinated rabbit blood in PBS buffer. After 15 min at 37°C, the unlysed blood cells were pelleted by centrifugation (5500× g, room temperature, 1 min). The hemolytic activity was determined by reading the optical density of the cell-free supernatant at 543 nm. The control culture supernatant served as the 100% hemolysis control, and relative percentage hemolysis values were calculated by comparison to this value.

Bacteria were cultured with 1/2× MIC of perilla oil to reach an OD _600nm_ of 2.5. Cells were collected by centrifugation, and washed three times in sterile PBS. Thereafter, bacteria were re-grown in fresh MHB to the post-exponential phase. The culture supernatants were collected and subjected to hemolysis assay as described above.

### Murine T-cell proliferation assay

The murine T-cell proliferation assay was performed according to the method described previously by Qiu et al. [31]. Overnight cultures of MSSA ATCC 29213, MRSA 2985, and MRSA 3701 in Roswell Park Memorial Institute Medium 1640 (RPMI 1640) (Invitrogen, CA, USA) were diluted 30-fold in 500 ml of prewarmed RPMI 1640 supplemented with 0.5% Tween-80; these cultures were incubated for 30 min at 37°C with constant shaking, and divided into 100 mL aliquots. Increasing concentrations of perilla oil (1/16, 1/8, 1/4 and 1/2× MIC) were added to the diluted bacterial suspensions before incubation for a further 4 h. Perilla oil-untreated *S. aureus* supernatants served as controls. Proteins secreted into the supernatants were filtered through a 0.2 µm pore-size filter and immediately analyzed as described below.

Specific-pathogen-free BALB/c mice (male, 6- to 8-weeks-old, weighing 18 to 22 g) were obtained from the Experimental Animal Center of Jilin University (Changchun, China). Spleen cell suspensions were prepared in RPMI 1640, washed and then resuspended in complete medium (RPMI 1640 media supplemented with 10% fetal bovine serum, 2 mM glutamine, 100 IU/ml penicillin, 100 IU/ml streptomycin, 15 mM HEPES and 50 µM 2-mercaptoethanol). A total of 10^6^ (150 µl) cells were dispensed into the wells of a 96-well tissue culture plate.

Cell proliferation was determined by the MTT assay. *S. aureus* culture supernatants (50 µl) were added to the tissue culture plates described above. After incubation at 37°C for 72 h with 5% CO_2_, 20 µl MTT (5 mg/ml) dissolved in PBS was added to each well, and the plate was incubated for a further 4 h at 37°C. The cells were collected by centrifugation for 10 min at 500×g. The pellet was redissolved in 150 µl DMSO at room temperature for 10 min, and the optical density at 570 nm was measured using a microplate reader (TECAN, Austria). The splenocytes' viability and number are represented by OD_ 570 nm_.

### Determination of proteolytic activity and extracellular protein concentration

Bacteria were grown in MHB supplemented with 0.5% Tween-80 in the absence or presence of increasing concentrations of perilla oil until reaching an OD_600 nm_ of 2.5, the culture supernatants were harvested by centrifugation.

For proteolytic activity assay, a 100 µl volume of supernatant was added to 1 ml of azocasein (Sigma-Aldrich) and incubated at 37°C for 1 h. Thereafter, 1 ml trichloroacetic acid (5%, w/v) was applied to terminate the reaction; undigested azocasein was allowed to precipitate for 30 min. The mixture was then centrifuged at 10,000×g for 10 min, and the absorbance of the supernatant was read at 328 nm. One unit of protease activity was defined as giving an absorbance of 0.001 after incubation for 1 h at 37°C.

The culture supernatants were precipitated by adding 100% trichloroacetic acid (Sigma) to a final concentration of 10%. After overnight incubation at 4°C, the precipitate was centrifuged at 15,000×*g* for 20 min at 4°C and finally washed three times with ice-cold (−20°C) ethanol. The aggregated proteins were dried by using a Speed-Vac for a few minutes. The protein extracts were dissolved in 0.5 ml of 0.1 M Tris.

The protein concentrations were determined with a Bio-Rad (Munich, Germany) protein assay kit according to the instructions of the manufacturer.

### SDS-PAGE and western blot assay

Equal amount of protein was subjected to Sodium dodecyl sulfate (SDS)-polyacrylamide (12%) gel electrophoresis run at 120 V. The gel was stained overnight in 0.1% Coomassie brilliant blue R250 with 25% isopropanol and 10% acetic acid.

For western blot assay, proteins were transferred to polyvinylidene fluoride membranes (Wako Pure Chemical Industries, Ltd, Osaka, Japan) using a semi-dry transfer cell (Bio-Rad, Munich, Germany). Membranes were incubated overnight at 4°C in 10% milk powder (a blocking reagent). The production of α-toxin, SEA, SEB, and TSST-1 in *S. aureus* was detected by incubation with indicated antibodies. Antibodies to α-toxin, SEA, and SEB were purchased from Sigma-Aldrich and diluted to 1∶10000, 1∶5000 and 1∶8000, respectively; a horseradish peroxidase-conjugated anti-rabbit antiserum (Sigma-Aldrich) diluted to 1∶4000 was then used as the secondary antibody. The antibody to TSST-1 (Santa Cruz Biotechnology, California, USA) was diluted to 1∶200 according to the manufacturer's recommendation, and a horseradish peroxidase-conjugated anti-mouse antiserum (Sigma-Aldrich) diluted to 1∶5000 was used as the secondary antibody. The membrane was treated with ECL substrate (GE Healthcare, Buckinghamshire, UK) according to the manufacturer's instructions, and exposed to x-ray film.

### RNA isolation and real-time RT-PCR

*S. aureus* strain ATCC 29213 was incubated with or without 0.2 µl/ml of perilla oil to the post-exponential growth phase (OD_600 nm_ of 2.5) as described in the hemolysis assay. Cells were harvested by centrifugation (5,000×g for 5 min at 4°C) and resuspended into TES buffer (10 mM Tris-Cl, 1 mM EDTA, 0.5% SDS) containing 100 µg/ml of lysostaphin (Sigma-Aldrich). Following incubation at 37°C for 10 min, a Qiagen RNeasy Maxi column was used to isolate total bacterial RNA, which was in accordance with the manufacturer's instructions. The contaminating DNA was removed using the optional on-column RNase-free DNase I step (Qiagen, Hilden, Germany). RNA concentrations were determined by reading the OD_260 nm_, and the RNA was loaded onto an RNase-free 2% agarose gel to test for generalized degradation. Equal amounts of RNA were reverse transcribed into cDNA using the Takara RNA PCR kit (AMV) Ver. 3.0 (Takara, Kyoto, Japan) according to the manufacturer's protocol. Genes names and primer sequences used in the real-time RT-PCR analysis are listed in Table 4. The real-time RT-PCR was performed using the 7000 Sequence Detection System (Applied Biosystems, Courtaboeuf, France) and SYBR Premix Ex Taq (Takara). Reaction mixtures were initially incubated for 30 s at 95°C, followed by 35 cycles of 5 s at 95°C, 30 s at 55°C, and 20 s at 72°C. Melt-curve analysis was also performed to assess PCR specificity, resulting in single primer-specific melting temperatures. All samples were analyzed in triplicate and the constitutively expressed gene for gyrase (*gyr*) was used as an endogenous control as described previously [32]. In this study, relative quantification based on the expression of a target gene versus the *gyr* gene was utilized to determine the transcript level changes between samples.

**Table 4:** Primers used for real-time RT-PCR.

Primer	Sequence	Location within gene
*gyr*-fw	5′- AGGTCCTGATTTCCC -3′	642–657
*gyr*-rv	5′- GAGCCTTATTCACTTGG -3′	814–831
*hla* -fw	5′-TTGGTGCAAATGTTTC-3′	485–501
*hla*-rv	5′-TCACTTTCCAGCCTACT-3′	569–586
*sea*-fw	5′-ATGGTGCTTATTATGGTTATC-3′	335–356
*sea*-rv	5′-CGTTTCCAAAGGTACTGTATT-3′	477–498
*seb*-fw	5′-TGTTCGGGTATTTGAAGATGG-3′	480–501
*seb*-rv	5′-CGTTTCATAAGGCGAGTTGTT-3′	612–633
*tst*-fw	5′-ACCCCTGTTCCCTTATCATC-3′	73–93
*tst*-rv	5′-AAAAGCGTCAGACCCACTAC-3′	159–180
*agrA-* fw	5′-TGATAATCCTTATGAGGTGCTT-3′	111–133
*agrA-* rv	5′-CACTGTGACTCGTAACGAAAA-3′	253–274

### Statistical analysis

SPSS 12.0 statistical software was used to analyze experimental data. The independent Student's t-test was used to determine statistical differences, and a *p* value of less than 0.05 was considered to be statistically significant.

## Supporting Information

Figure S1 Scanning electron micrographs of *S. aureus* ATCC 29213 after treatment with graded subinhibitory concentrations of perilla oil to the post-exponential growth phase. (A) perilla oil-free culture; (B) *S. aureus* cultured with 1/2 MIC of perilla oil; (C) *S. aureus* cultured with 1/4 MIC of perilla oil; (D) *S. aureus* cultured with 1/8 MIC of perilla oil; (E) *S. aureus* cultured with 1/16 MIC of perilla oil.
(TIF)
