# Supplementary figures and images for: Subinhibitory Concentrations of Perilla Oil Affect the Expression of Secreted Virulence Factor Genes in Staphylococcus aureus
Source: PLoS One. 2011 Jan 19;6(1):e16160. doi: 10.1371/journal.pone.0016160 (PMC3023776; doi:10.1371/journal.pone.0016160)

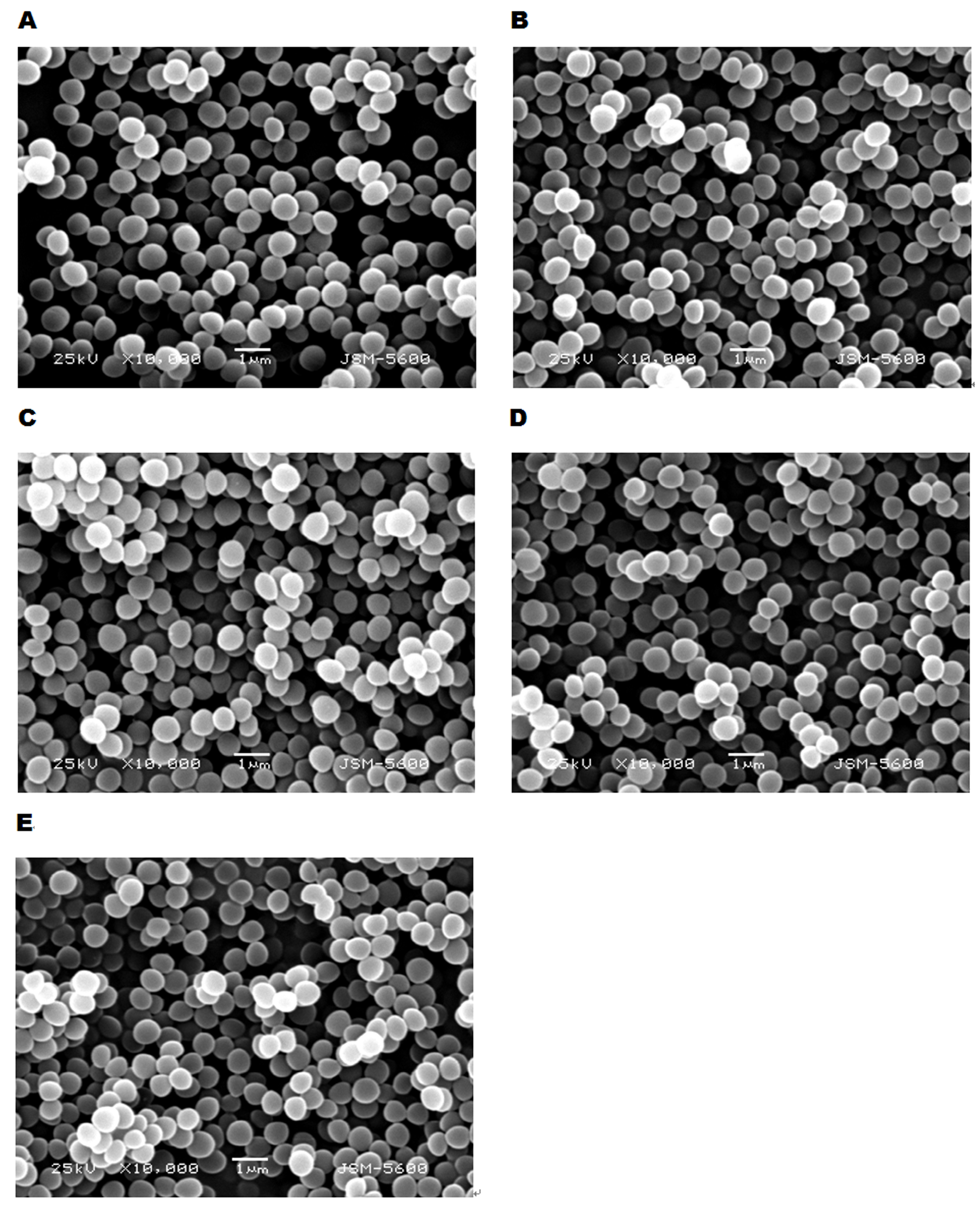

Supplement: Figure S1 — Scanning electron micrographs of S. aureus ATCC 29213 after treatment with graded subinhibitory concentrations of perilla oil to the post-exponential growth phase. (A) perilla oil-free culture; (B) S. aureus cultured with 1/2 MIC of perilla oil; (C) S. aureus cultured with 1/4 MIC of perilla oil; (D) S. aureus cultured with 1/8 MIC of perilla oil; (E) S. aureus cultured with 1/16 MIC of perilla oil. (TIF) [file pone.0016160.s001.tif]
